# Longitudinal Assessment of EGFR and MET Transcriptional Activity Using Cell-Free Chromatin Immunoprecipitation in Two Patients with EGFR-Mutated Non-Small Cell Lung Cancer

**DOI:** 10.3390/cancers18193097

**Published:** 2026-09-23

**Authors:** Emma Pedersen, Christoffer Trier Maansson, Peter Meldgaard, Boe Sandahl Sorensen

**Affiliations:** 1 Department of Clinical Biochemistry, Aarhus University Hospital, 8200 Aarhus, Denmarkctm@clin.au.dk (C.T.M.); 2 Department of Clinical Medicine, Aarhus University, 8000 Aarhus, Denmark; 3 Department of Oncology, Aarhus University Hospital, 8200 Aarhus, Denmark; petemeld@rm.dk

**Keywords:** epigenetics, osimertinib, crizotinib, liquid biopsies, cell-free chromatin immunoprecipitation, resistance, NSCLC

## Abstract

**Background/Objectives:** *MET* amplification represents the most common bypass resistance mechanism to EGFR tyrosine kinase inhibitor (TKI) treatment in EGFR-mutated non-small cell lung cancer (NSCLC). Combination therapy with the EGFR-TKI, osimertinib, and the MET-TKI, crizotinib, has demonstrated clinical efficacy in overcoming this resistance. However, assessing MET transcriptional activity during treatment remains challenging. **Methods:** Cell-free chromatin immunoprecipitation (cfChIP) targeting H3 lysine 36 trimethylation (H3K36me3) was employed to investigate the transcriptional activity of the *EGFR* and *MET* genes in serial liquid biopsies obtained from two patients with EGFR-mutated NSCLC. Droplet digital PCR (ddPCR) was used to quantify circulating tumor DNA (ctDNA) and cfChIP enrichment levels. **Results:** In PT1, H3K36me3 m*EGFR* and *MET* cfChIP enrichment increased during disease progression, with *MET* enrichment increasing further following the initiation of crizotinib/osimertinib combination therapy, during which the patient experienced a partial response. In PT2, *MET* amplification was detected in a liquid biopsy, but *MET* cfChIP enrichment remained low throughout the treatment course, and the patient did not respond to crizotinib. **Conclusions:** This two-patient feasibility study illustrates the potential of cfChIP-ddPCR for the longitudinal assessment of *EGFR* and *MET* transcriptional activity in liquid biopsies. These observations warrant further investigation of cfChIP as an approach for monitoring transcriptional activity during osimertinib/crizotinib treatment.

## Simple Summary

Lung cancer patients with EGFR mutations often develop resistance to the drug osimertinib when another gene, MET, becomes overactive. Combining osimertinib with the MET-blocking drug crizotinib can help overcome this resistance, but monitoring MET activity during treatment remains challenging. Using a novel blood test called cfChIP, we tracked EGFR and MET gene activity in serial blood samples from two lung cancer patients. In one patient, increased *MET* cfChIP enrichment was observed during progression and following crizotinib treatment, while a second patient with *MET* amplification had persistently low *MET* cfChIP enrichment and did not respond to crizotinib. These initial observations warrant further research into whether cfChIP can be used as a blood-based approach to assess gene activity in patients receiving osimertinib and crizotinib.

## 1. Introduction

Lung cancer, with 2.5 million new cases and 1.8 million deaths reported in 2022, remains the leading cause of cancer incidence and mortality [1,2]. Lung cancer is broadly classified into small cell lung cancer and non-small cell lung cancer (NSCLC), the latter being the most common form, accounting for 85% of cases [3]. Adenocarcinoma is the most frequent NSCLC subtype and approximately 12–17% harbor activating mutations in the epidermal growth factor receptor (EGFR) [4,5]. The most common activating mutations include in-frame deletions of exon 19 (ex19del) and the L858R mutation in exon 21 [6]. Osimertinib, the third-generation EGFR tyrosine kinase inhibitor (EGFR-TKI), is the standard first-line treatment for advanced EGFR-mutated NSCLC in most countries [7,8]. However, acquired resistance is inevitable, with MET amplification being the most common EGFR bypass mechanism [9]. This has led to combination therapy with osimertinib and novel MET-TKIs, such as savolitinib, with promising results in early-phase clinical studies [10,11,12]. Crizotinib, which inhibits MET among other kinases, has also demonstrated benefit in some case studies [13,14]. However, treatment response to MET-TKIs appears to differ between patients, underscoring the need to identify predictive biomarkers.

Minimally invasive liquid biopsies are widely investigated, as they allow longitudinal disease monitoring through repeated sampling without the complications and patient discomfort associated with traditional tissue biopsies [15,16,17,18,19,20]. From a blood sample, tumor-derived cell-free DNA (cfDNA), referred to as circulating tumor DNA (ctDNA), provides information on tumor genetics in cancer patients [19,21]. ctDNA has emerged as a biomarker in *EGFR*-positive NSCLC to track tumor burden and predict both TKI response and disease [22,23]. Clearance of ctDNA during osimertinib treatment has been associated with early response leading to longer progression-free (PFS) and overall survival (OS), whereas elevated *EGFR* mutant allele frequency (MAF) has been associated with disease progression [24,25]. The utility of ctDNA as a biomarker has primarily been confined to the detection of gene alterations, but recent technological advancements have enabled the ability to detect the epigenetic properties of cfDNA [26]. Cell-free chromatin immunoprecipitation (cfChIP) utilizes post-translational histone modifications on cell-free nucleosomes in the bloodstream and reflects gene expression in the cell from which it originated [27,28,29]. This includes the trimethylation of lysine 36 on histone H3 (H3K36me3), which is associated with active gene transcription [27,30,31,32]. cfChIP targeting H3K36me3 enables the selective immunoprecipitation of cell-free nucleosomes in plasma bearing this modification, thereby reflecting transcriptionally active genes. Previously, our lab demonstrated that H3K36me3-cfChIP followed by droplet digital PCR (ddPCR) quantification results in the enrichment of mutant *EGFR* fragments compared to *EGFR* wild type, consistent with the active transcription of the *EGFR* mutant allele in the tumor [27]. In this study, we expanded the application of the cfChIP methodology to longitudinally monitor the transcriptional activity of mutant *EGFR* (mEGFR) and *MET* in patients treated with osimertinib and crizotinib. The aim of this proof-of-concept study was to explore the feasibility of using cfChIP to assess gene-associated transcriptional activity longitudinally in patients treated with osimertinib. The observations provide a rationale for further investigation of the approach in larger prospective studies.

## 2. Materials and Methods

### 2.1. Blood Plasma

The study was conducted in compliance with the Declaration of Helsinki and was approved by the Central Denmark Region Committee on Biomedical Research Ethics (No. 1-16-02-211-16). Informed consent was obtained from all individuals involved in the study. The included patients were diagnosed with stage IV EGFR-mutated NSCLC and received first-line osimertinib therapy. The patients developed resistance through MET amplification after which they received crizotinib/osimertinib combination therapy. Serial blood samples were collected before and after initiation of the combination therapy. Peripheral blood was collected into Streck or EDTA-containing tubes and centrifuged within 2 h at 1400 *g* for 15 min at room temperature. Aliquoted plasma was stored at −80 °C until use.

### 2.2. Next-Generation Sequencing of cfDNA

Cell-free DNA was purified from plasma using the AVENIO cfDNA Isolation Kit (Roche, Basel, Switzerland) and sequenced using the AVENIO ctDNA Surveillance Kit (Roche). The next-generation sequencing (NGS) library was sequenced using a NextSeq 500 and data were processed using the AVENIO Oncology Analysis Software v. 2.0.0 (Roche). For this study, we only focused on the detection of *MET* amplification, which was defined as a CNV score > 5.

### 2.3. Cell-Free Chromatin Immunoprecipitation

cfChIP was executed as outlined in Maansson et al. [33]. Briefly, plasma was cleared of circulating antibodies using empty protein A/G magnetic beads (Thermo Fisher Scientific, Waltham, MA, USA) and subsequently mixed with anti-H3K36me3-coated protein A/G magnetic beads followed by incubation O/N at 4 °C. Beads were washed and precipitated DNA was eluted. cfChIP samples were purified using the Apostle MiniMax High Efficiency cfDNA Isolation kit (Apostle Bio, Pleasanton, CA, USA). Input cfDNA samples were purified with the Apostle MiniMax High Efficiency cfDNA Isolation kit or the AVENIO ctDNA Isolation Kit V2 (Roche).

### 2.4. Cell Cultures

The study uses ChIP and input DNA samples extracted from the lung adenocarcinoma cell lines, including parental HCC827 (RRID: CVCL_2063, ATCC, LCG Standards, Wesel, Germany), its MET-amplified subclone (HCC827-MET) described in [34], and A549 (RRID: CVCL_0023, ATCC, LCG Standards). Cell culture, chromatin isolation, chromatin immunoprecipitation (ChIP) and DNA purification were all performed as described by Maansson et al. [33].

### 2.5. Droplet Digital PCR

Droplet digital PCR was performed using the QX200 AutoDG Droplet Digital PCR System (Bio-Rad, Hercules, CA, USA) and Bio-Rad’s mutation assays for *EGFR E19del* (Bio-Rad, Assay ID dHsaMVD2010039) and *MET* WT (Bio-Rad, Assay ID dHsaIS2503879, Table A1). The MET WT assay was supplied as part of a duplex assay containing primer/probe sets for both wild-type and mutant MET. However, only the wild-type MET assay was used for the analyses presented in this study.

The experiments were run in duplicates where each well contained 11 ddPCR supermix for probes (no dUTP), 1 µL of each primer (Table A2) and probe (Table A3) and 5–10 µL cfChIP or input sample for a total volume of 22 µL. Droplets were generated using the QX200 automated droplet generator (Bio-Rad) and PCR amplification was performed on a thermocycler (Eppendorf SE, Hamburg, Germany) with the following setup: initial denaturation at 95 °C for 10 min, 40 PCR cycles of 30 s denaturation at 94 °C and 1 min annealing at 55 °C or 58 °C, 10 min at 98 °C followed by rest at 4 °C O/N. Droplets were read using the QX200 Droplet Reader (Bio-Rad) and data were analyzed using Bio-Rad’s Quantasoft^TM^ software version 1.7.4. Non-template controls containing HyPure water were used to establish the threshold for positive droplets. Mutant allele frequencies were calculated as the number of droplets positive for the mutant variant compared to the total number of WT and variant positive droplets. ChIP enrichment was estimated by counting the number of positive cfChIP droplets and adjusting for differences in plasma used for cfChIP, elution volumes and sample volumes used for ddPCR. cfChIP enrichment was used as a measure of the total transcriptional activity of the target gene, irrespective of whether this activity resulted from increased transcription per gene copy or from gene amplification. Accordingly, cfChIP enrichment was not adjusted for gene copy number.

## 3. Results

### 3.1. Presentation of Patient Cases

The first patient (PT1, Figure 1 top) was a 29-year-old woman, never-smoker with Li–Fraumeni syndrome, carrying a germline TP53 mutation, predisposing to multiple cancers [35]. She was diagnosed with stage IV EGFR-ex19del-mutated NSCLC with liver and bone metastases. First-line osimertinib induced a response for two years, after which progression was treated with chemotherapy (carboplatin/pemetrexed) in combination with osimertinib, resulting in a further six-month response. At subsequent progression, MET amplification was detected in both liquid and tissue biopsies from a new liver metastasis. Crizotinib was therefore added to osimertinib, resulting in a response in all tumor lesions within one month. The second patient (PT2, Figure 1 bottom) was a 36-year-old never-smoker with stage IV EGFR-ex19del-mutated NSCLC with liver, bone, and brain metastases. First-line osimertinib induced a response for nearly 1.5 years. At 16 months, progression was observed in a single liver metastasis, with MET amplification detected in a liquid biopsy. EGFR-ex19del was confirmed in the liver metastasis, but no additional genetic alterations were identified. Crizotinib was subsequently added to continued osimertinib treatment, but the patient showed continuous disease progression and no response to the combination. Crizotinib was discontinued, and treatment was changed to radiotherapy and chemotherapy (carboplatin/vinorelbine) while osimertinib was continued, resulting in a partial response within two months.

### 3.2. Evaluating EGFR and MET Transcription in Liquid Biopsies

Both patients received osimertinib and subsequently had *MET* amplification detected in liquid biopsies. We therefore assessed *EGFR* and *MET* cfChIP enrichment longitudinally to characterize gene-associated transcriptional activity during their treatment courses. We developed ChIP-ddPCR assays to quantify *EGFR* and *MET* gene activity and validated them in multiple cell lines (see Figure A1 and Figure A2, Appendix A).

H3K36me3 cfChIP-ddPCR was performed on longitudinal plasma samples from PT1 and PT2. To confirm that cfChIP enrichment reflected gene activity in the cells of origin, we quantified the active gene *ACTG1* and the lowly expressed gene *DNAJC5G* (Figure 2A). Blood cells are the primary source of cfDNA [36] and show high *ACTG1* but low *DNAJC5G* expression (Figure A3). Consistent with this, *ACTG1* showed clear cfChIP enrichment (Figure 2B), demonstrating that cfChIP can detect transcriptionally active genes in plasma.

To assess *EGFR* and *MET* gene-associated transcriptional activity, we quantified m*EGFR* and *MET* cfChIP enrichment in serial plasma samples. Before first-line osimertinib, PT1 had a high ctDNA fraction, reflected by an *EGFR* MAF of 54.9% (Figure 3A), and m*EGFR* showed 1.5-fold higher cfChIP enrichment than *ACTG1*. Following osimertinib treatment, m*EGFR* became undetectable in the plasma and cfChIP samples, coinciding with partial response (PR). At progression (22 months), m*EGFR* reappeared in plasma, while subsequent chemotherapy reduced the ctDNA levels. At the second progression (30 months), m*EGFR* cfChIP enrichment increased to 1.2-fold relative to *ACTG1*, accompanied by an *EGFR* MAF of 32.5%. At this time, *MET* amplification was detected and *MET* cfChIP enrichment increased to 2.1-fold relative to *ACTG1*, corresponding to a 6.7-fold increase in *MET* enrichment compared with baseline. Following the addition of crizotinib to osimertinib, *MET* cfChIP enrichment increased further to 3.3-fold relative to *ACTG1* (32 months), while m*EGFR* enrichment and *EGFR* MAF decreased to 4.3%, coinciding with radiological PR. Subsequent samples showed declining ctDNA levels, low m*EGFR* MAF, and reduced *MET* enrichment.

In PT2, partial response to osimertinib after four months was reflected by a low m*EGFR* MAF of 0.4% (Figure 3B). The m*EGFR* MAF increased to 5.6% at 13 months, and *MET* amplification was detected in a liquid biopsy at 15 months. At progression (18 months), the m*EGFR* MAF had increased to 20.7%, while cfChIP showed only moderate m*EGFR* enrichment. Despite the detected *MET* amplification, *MET* cfChIP enrichment remained low, with no increase relative to baseline. The patient subsequently received crizotinib but did not respond, whereas subsequent chemotherapy resulted in a partial response, accompanied by a decrease in the m*EGFR* MAF to 5.2% and later 0.4%.

## 4. Discussion

In this study, we investigated two patients with EGFR-mutated NSCLC in whom *MET* amplification was detected by a liquid biopsy as a potential resistance mechanism to first-line osimertinib. The aim was to explore whether cfChIP can provide additional information on gene transcriptional activity via serial liquid biopsies from patients treated with osimertinib. Analysis of the serial plasma samples with cfChIP ddPCR revealed increased m*EGFR* and *MET* enrichment in PT1, who responded to crizotinib/osimertinib combination therapy, both at disease progression on osimertinib and following the initiation of combination therapy. While m*EGFR* fragments were presumed exclusive to tumor cells, *MET* fragments can also originate from healthy cells, and the *MET* cfChIP signal has therefore not been established as tumor-specific. Consequently, increased *MET* cfChIP enrichment cannot by itself be interpreted as increased *MET* transcriptional activity specifically in the tumor. Nevertheless, in PT1, *MET* cfChIP enrichment increased at progression compared with baseline. The high ctDNA fraction at baseline (54.9%) with only minor *MET* enrichment, followed by increased ctDNA and *MET* enrichment at progression, provides a case-based observation compatible with increased *MET* transcriptional activity occurring during disease progression, although the tumor origin of the increased *MET* signal cannot be established from these data alone.

In PT2, *MET* amplification was detected in a liquid biopsy, while *MET* cfChIP enrichment remained low throughout the treatment course despite an increase in the ctDNA fraction from 0.4% to 20.7%. The *MET* amplification was detected in a liquid biopsy but not in the liver metastasis, and the possibility that the amplification originated from another tumor site cannot therefore be excluded. Following crizotinib treatment, the patient did not experience a response, whereas subsequent chemotherapy resulted in a partial response and a major reduction in the ctDNA fraction. The contrasting longitudinal observations in the two patients are of interest but do not establish a relationship between *MET* transcriptional activity and the response to MET inhibition. In particular, the cfChIP-*MET* signal has not been shown to measure functional MET dependency, and the absence of increased *MET* cfChIP enrichment cannot be used to determine whether the tumor was dependent on MET signaling.

The cfChIP method reflects the overall transcriptional activity of a given gene and does not distinguish increased transcription per gene copy from increased transcription resulting from gene amplification. In the present study, the objective was therefore to quantify the total gene-associated transcriptional activity in plasma rather than distinguish between these underlying mechanisms. Because both mechanisms are expected to result in increased gene expression and downstream biological activity, the cfChIP results were not adjusted for gene copy number.

Previous studies have reported associations between MET-related molecular characteristics and the response to MET-directed therapies. The phase 1b TATTON study of osimertinib in combination with the novel MET-TKI, savolitinib, indicated an increased effect in patients with *MET* copy number ≥ 10 in the tumor [12]. In addition, another phase 1 trial evaluating the combination of the third-generation EGFR-TKI, lazertinib, and the novel bispecific EGFR/MET inhibitor, amivantamab, also demonstrated an association between treatment response and high *EGFR* and/or *MET* expression in the tumor [37]. In the present study, cfChIP-ddPCR provided a minimally invasive approach for measuring *EGFR* and *MET* cfChIP enrichment longitudinally. The biological interpretation of *MET* cfChIP enrichment remains challenging, particularly with respect to its tumor specificity and relationship to functional MET signaling.

The primary limitation of this study is the very small sample size, comprising only two patients with contrasting clinical trajectories. In addition, PT1 had Li–Fraumeni syndrome, a condition associated with predisposition to multiple primary malignancies; as this patient had a prior breast cancer, it cannot be fully excluded that cfDNA signals attributed to the NSCLC were influenced by cfDNA derived from another tumor source. The *MET* amplification in PT2 was detected in the liquid biopsy but not in the liver metastasis biopsy, further limiting interpretation of the origin and biological significance of the *MET* signal. These limitations preclude conclusions regarding the tumor specificity or functional significance of cfChIP *MET* enrichment, as well as its potential clinical relevance for MET-targeted treatment. Rather, these two cases provide hypothesis-generating observations illustrating the biological information that may be obtained by assessing gene-associated transcriptional activity longitudinally in liquid biopsies and support further investigation in appropriately powered studies.

## 5. Conclusions

This proof-of-concept study illustrates that, in these two patients, MET amplification did not necessarily correspond to increased MET transcriptional activity and that elevated MET transcriptional activity was observed in the patient who responded to crizotinib. These findings provide a rationale for new prospective studies to further investigate longitudinal cfChIP-derived transcriptional activity during osimertinib/crizotinib combination therapy and establish its clinical relevance.

## Figures and Tables

**Figure 1:**
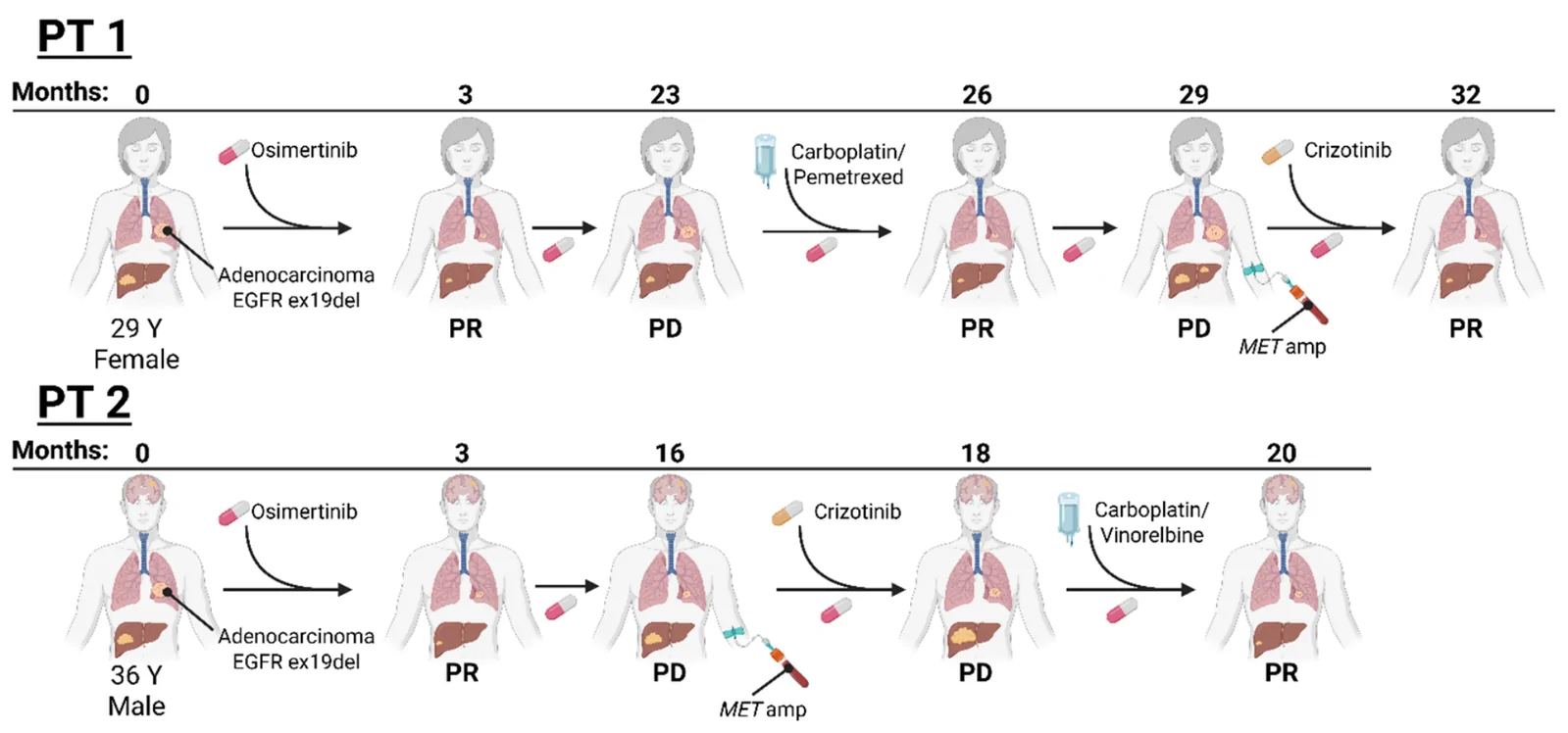
Overview of clinical cases. PT 1, 29 y female, (**top**) and PT 2, 36 y male, (**bottom**) are two *EGFR*-positive non-small cell lung cancer patients who received osimertinib as first-line treatment and subsequently developed *MET* amplification (amp) detected in a liquid biopsy. Each panel represents a schematic overview of the disease course and the treatments each patient received. PR: partial response, PD: progressive disease. Created with biorender.com.

**Figure 2:**
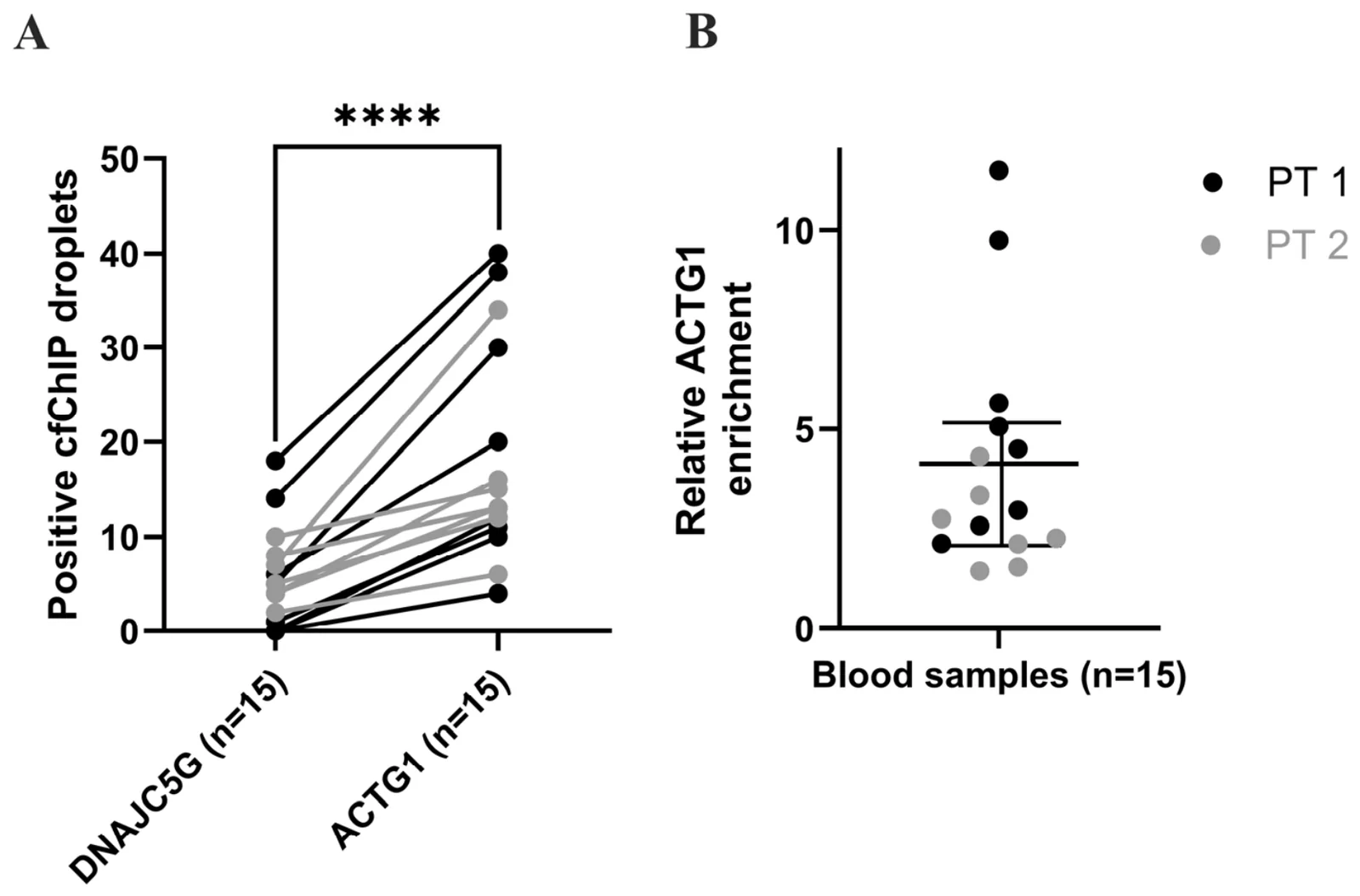
cfChIP enrichment of ACTG1 and DNAJC5G. (**A**) Paired positive DNAJC5G and ACTG1 droplets in cfChIP samples from PT1 and PT2. Statistical significance was tested with a paired *t*-test, **** two-tailed *p*-value < 0.0001. (**B**) Relative cfChIP enrichment of ACTG1 was calculated as the log2-transformed ratio between ACTG1 and DNAJC5G positive droplets + 1 pr. mL plasma in the cfChIP sample. Dots represent individual blood samples, the lines display the average and error bars show ±95% CI.

**Figure 3:**
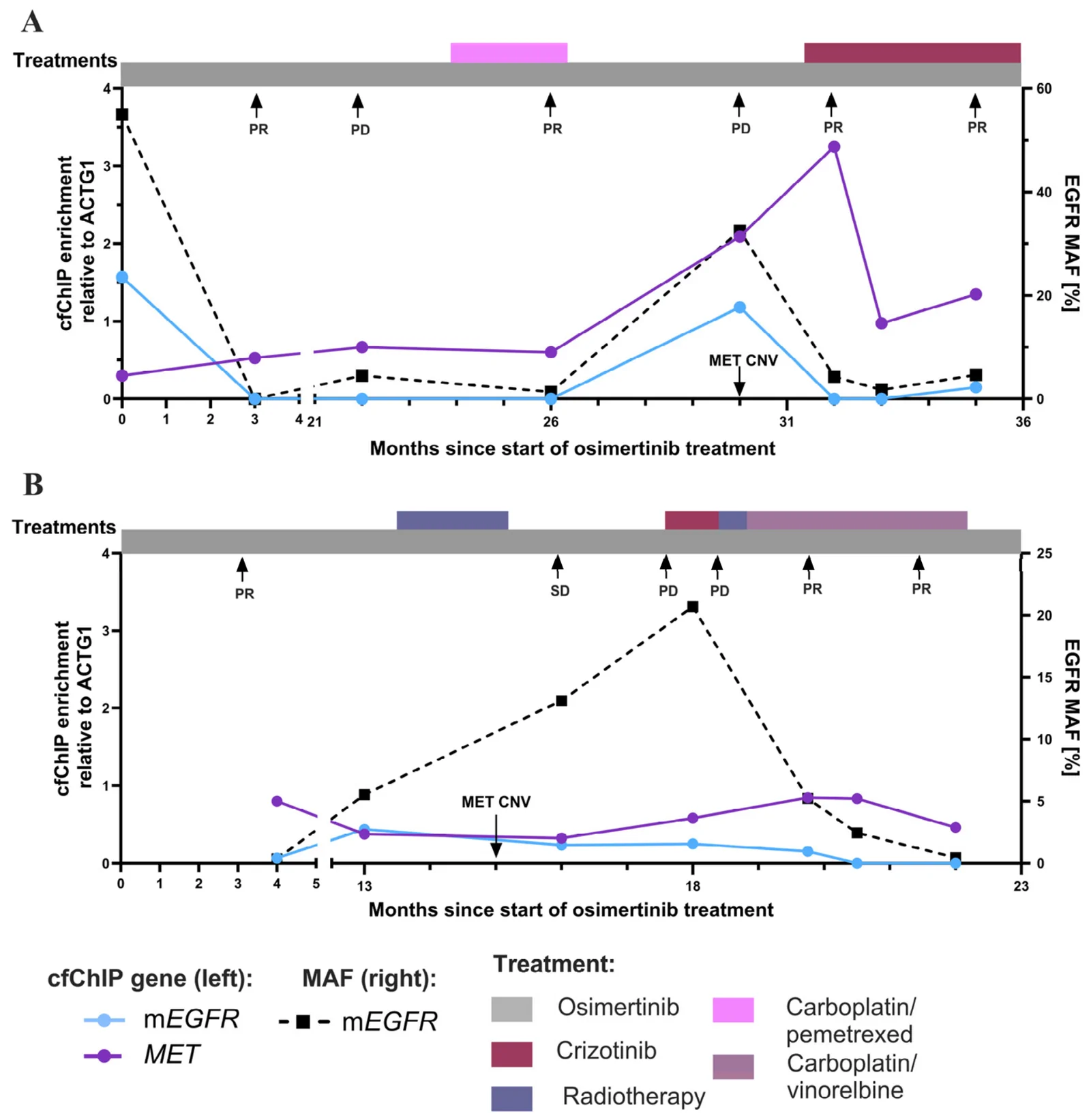
cfChIP-ddPCR analysis of EGFR and MET transcription in the two patient cases. mEGFR and MET enrichment is shown on the left Y axis as positive cfChIP droplets normalized to ACTG1. The EGFR mutant allele frequency (MAF) is plotted on the right Y axis. Above each plot, the treatment course is displayed. Arrows indicate clinical evaluation at routine visits. (**A**) PT1 and (**B**) PT2. CNV: copy number variation, PD: progressive disease, PR: partial response, SD: stable disease.

**Figure A1:**
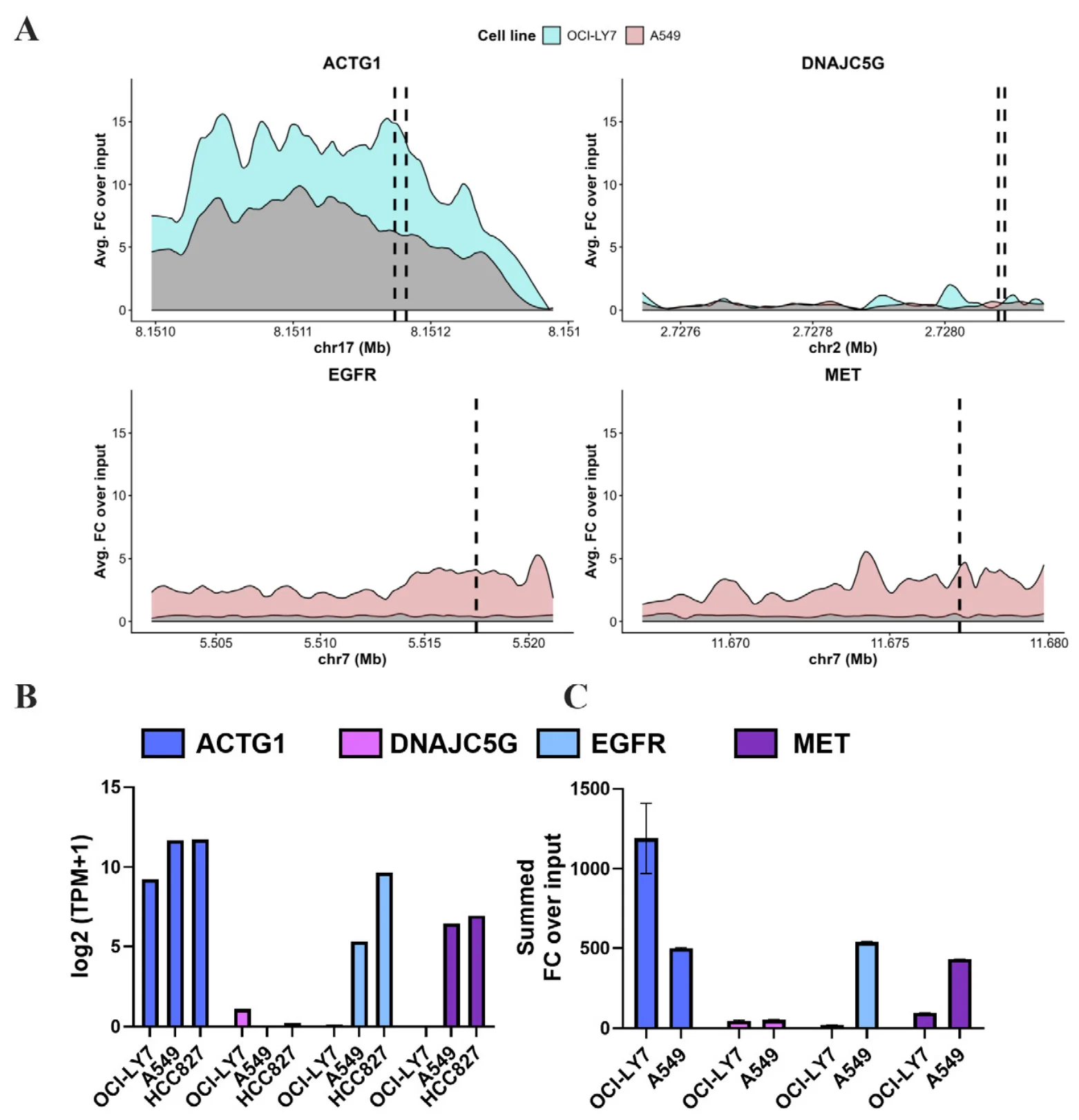
Comparison of H3K36me3 distribution and gene expression. (**A**) The average fold change over input across the genomic position. *ACTG1* (chr17:81509971-81512891), *DNAJC5G* (chr2:27275421-27281499), *EGFR* (chr7:55019021-55211628) and *MET* (chr7:116672390-116798385). Dashed lines mark the location of the ddPCR primer amplicons used for ChIP analysis. (**B**) RNA expression of *ACTG1*, *DNAJC5G*, *EGFR* and *MET* in OCI-LY7, A549 and HCC827 cells. (**C**) Average fold change over input, summarized across the amplicon regions. The upper and lower error bars reflect the sample-specific fold change over input.

**Figure A2:**
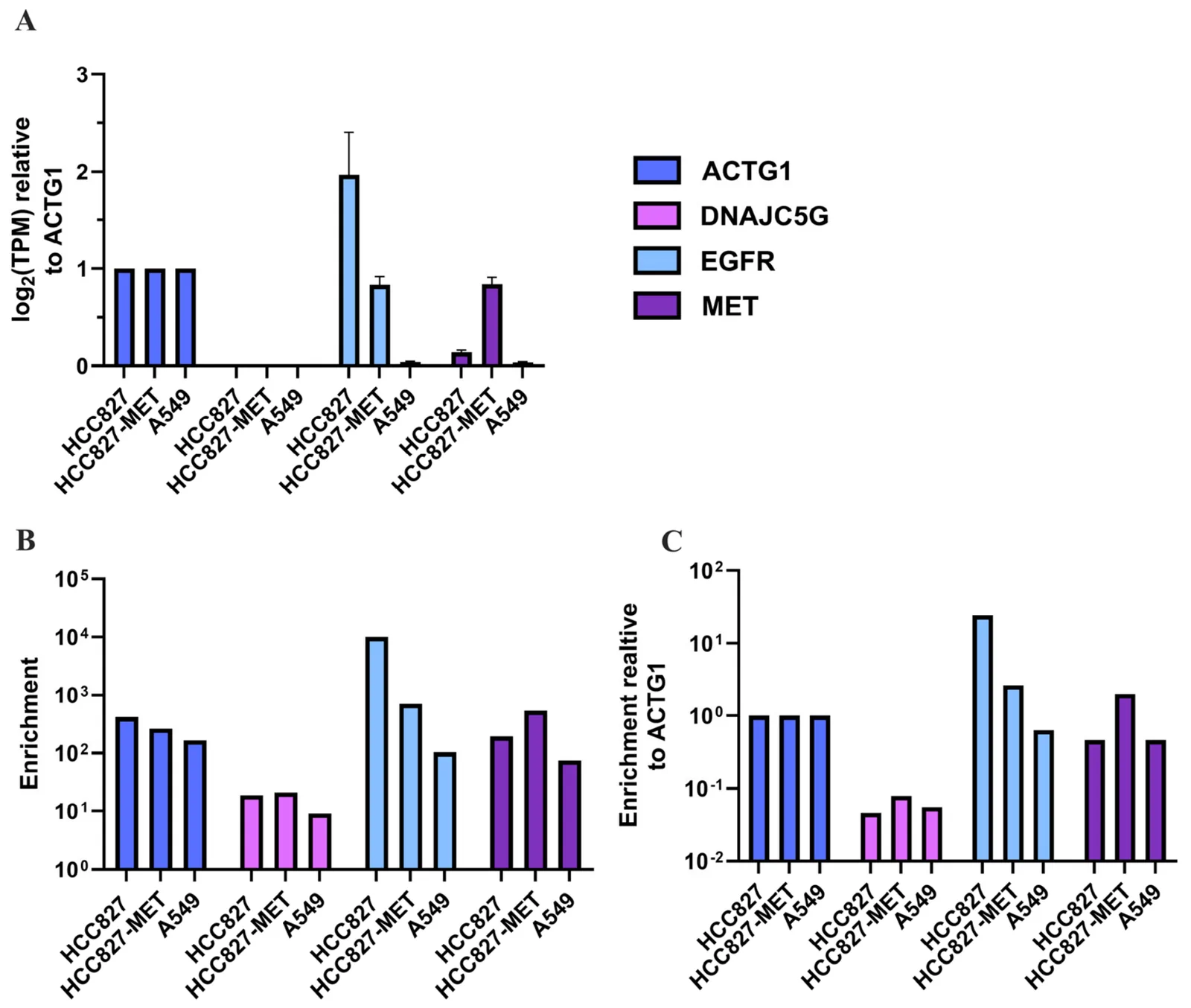
Cell line-based validation of ChIP-ddPCR for analyzing *MET* and *EGFR* transcription. (**A**) RNA-seq-based expression levels of *EGFR*, *MET* and *DNAJC5G* gene expression levels in parental HCC827, *MET*-amplified HCC827-MET and A549 cells, plotted as log_2_(TPM) relative to *ACTG1*. (**B**) Positive droplets of *EGFR*, *MET*, *ACTG1* and *DNAJC5G* in ChIP samples in parental HCC827, HCC827-MET and A549 cells. (**C**) Relative enrichment of *MET*, *EGFR* and *DNAJC5G* based on the number of positive ChIP droplets relative to *ACTG1* in parental HCC827, HCC827-MET and A549 cells.

**Figure A3:**
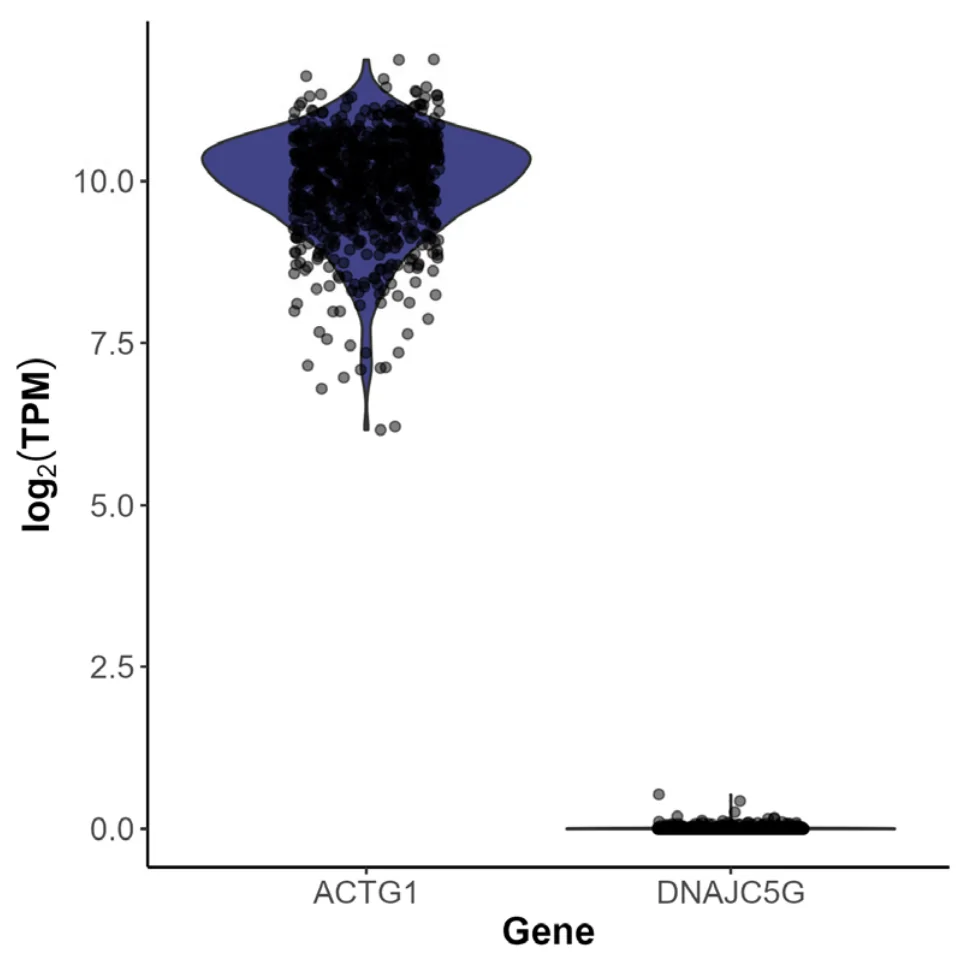
ACTG1 and DNAJC5G expression levels from GTEx whole blood RNA-seq data plotted as log_2_(TPM).

## Data Availability

The data presented in this study are available on request from the corresponding author. The data are not publicly available due to privacy and ethical restrictions.

## Abbreviations

The following abbreviations are used in this manuscript:

amp	Amplification
cfChIP	Cell-free chromatin immunoprecipitation
cfDNA	Cell-free DNA
ChIP	Chromatin immunoprecipitation
CNV	Copy number variation
ctDNA	Circulating tumor DNA
ddPCR	Droplet digital PCR
EGFR	Epidermal growth factor receptor
Ex19del	Deletion of exon 19
H3K36me3	H3 lysine 36 trimethylation
MAF	Mutational allele fraction
mEGFR	Mutant EGFR
NGS	Next-generation sequencing
NSCLC	Non-small cell lung cancer
OS	Overall survival
PD	Progressive disease
PR	Partial response
PFS	Progression-free survival
SD	Stable disease
TKI	Tyrosine kinase inhibitor

## Appendix A. Validation of ChIP ddPCR Assay

H3K36me3 ChIP-Seq data from A549 (access: ENCSR000AUL) and OCI-LY7 (access: ENCSR243AIS) cell lines provided by ENCODE were used to evaluate the H3K36me3 distribution in genes of interest. The housekeeping gene, *ACTG1*, demonstrated gene-wide H3K36me3 enrichment in both cell lines (Figure A1A) whereas the weakly transcribed gene, *DNAJC5G*, had no H3K36me3 enrichment consistent with RNA expression levels from the DepMap 24Q4 expression dataset (Figure A1B). Similarly, the H3K36me3 enrichment across EGFR and MET was higher in A549 cells compared to OCI-LY7 cells, consistent with the higher expression of both genes in A549. We designed amplicons in the 3′ end of these genes to be used in ChIP assays. The summarized enrichment in these amplicon regions (Figure A1C) is consistent with the RNA expression levels in the genes of interest, highlighting that ChIP-ddPCR enrichment reflects RNA expression.

We performed H3K36me3 ChIP followed by ddPCR on the following cell lines: HCC827, MET-amplified HCC827 (HCC827-MET), and A549. RNA-seq [33] has revealed these cells have markedly different expression of *EGFR* and *MET* (Figure A2A). Quantifying the ChIP enrichment in these genes revealed high *ACTG1* enrichment and very low *DNAJC5G* enrichment (Figure A2B). Importantly, HCC827-MET has elevated *MET* enrichment compared to HCC827 and A549 (Figure A2B,C). Similarly, HCC827 has elevated *EGFR* enrichment compared to A549 and HCC827-MET. These findings illustrate anti-H3K36me3 ChIP-ddPCR enables quantification of *MET* and *EGFR* transcriptional activity and support the use of ChIP on cfDNA to study gene expression in the cell of origin.

## Appendix B

**Table A1:** Bio-Rad’s mutation assays used for ddPCR.

Gene	Coding Change	Amino Acid	Bio-Rad Assay	Amplicon Size (bp)	Fluorophore
EGFR	c.2235_2249del15	p.E746_A750del	dHsaMVD2010039	75	FAM/HEX
MET	c.3029C > T	p.T1010I	dHsaIS2503879	70	HEX

**Table A2:** Primer names, sequences and melting temperatures.

Name	Sequence 5′-3′	Tm (°C)	Amplicon Size (bp)
DNJAC5G_fwd	GCGATATCACCACCAAGAGCCT	64.8	98
DNAJC5G_rev	GGTACAGAGAGTTGCAACAAGGG	62.3	
ACTG1_fwd	GTTTCTTTCGCTGTTCCAGG	59.9	84
ACTG1_rev	GCAGGCAGAAACCAAATGAG	60.8	

**Table A3:** Probe names, sequences and melting temperatures.

Name	Sequence 5′-3′	Tm (°C)
DNAJC5G	FAM-ATCCTGTCCCTGGAGCTGCTGCACC-BHQ1	70.9
ACTG1	HEX-CCCGGCATTTCCTCCCTGAAGCCTCC-BHQ1	65.9
